# Beyond New Neurons in the Adult Hippocampus: Imipramine Acts as a Pro-Astrogliogenic Factor and Rescues Cognitive Impairments Induced by Stress Exposure

**DOI:** 10.3390/cells11030390

**Published:** 2022-01-24

**Authors:** Ana R. Machado-Santos, Eduardo Loureiro-Campos, Patrícia Patrício, Bruna Araújo, Nuno Dinis Alves, António Mateus-Pinheiro, Joana Sofia Correia, Mónica Morais, João M. Bessa, Nuno Sousa, Ana J. Rodrigues, João Filipe Oliveira, Luísa Pinto

**Affiliations:** 1 Life and Health Sciences Research Institute (ICVS), School of Medicine, University of Minho, 4710-057 Braga, Portugal; anaritasantus@gmail.com (A.R.M.-S.); id7472@alunos.uminho.pt (E.L.-C.); patriciapatricio@med.uminho.pt (P.P.); pg34788@alunos.uminho.pt (B.A.); nunodinisalves@gmail.com (N.D.A.); antoniomateuspinheiro@gmail.com (A.M.-P.); id8212@alunos.uminho.pt (J.S.C.); monica.dias-morais@pasteur.fr (M.M.); joaobessa@med.uminho.pt (J.M.B.); njcsousa@med.uminho.pt (N.S.); ajrodrigues@med.uminho.pt (A.J.R.); 2 ICVS/3B’s—PT Government Associate Laboratory, Braga/Guimarães, Portugal; 3 IPCA-EST-2Ai, Polytechnic Institute of Cávado and Ave, Applied Artificial Intelligence Laboratory, Campus of IPCA, 4750-810 Barcelos, Portugal

**Keywords:** astrocytes, hippocampus, dentate gyrus, astrogliogenesis, chronic stress, antidepressants

## Abstract

Depression is a prevalent, socially burdensome disease. Different studies have demonstrated the important role of astrocytes in the pathophysiology of depression as modulators of neurotransmission and neurovascular coupling. This is evidenced by astrocyte impairments observed in brains of depressed patients and the appearance of depressive-like behaviors upon astrocytic dysfunctions in animal models. However, little is known about the importance of de novo generated astrocytes in the mammalian brain and in particular its possible involvement in the precipitation of depression and in the therapeutic actions of current antidepressants (ADs). Therefore, we studied the modulation of astrocytes and adult astrogliogenesis in the hippocampal dentate gyrus (DG) of rats exposed to an unpredictable chronic mild stress (uCMS) protocol, untreated and treated for two weeks with antidepressants—fluoxetine and imipramine. Our results show that adult astrogliogenesis in the DG is modulated by stress and imipramine. This study reveals that distinct classes of ADs impact differently in the astrogliogenic process, showing different cellular mechanisms relevant to the recovery from behavioral deficits induced by chronic stress exposure. As such, in addition to those resident, the newborn astrocytes in the hippocampal DG might also be promising therapeutic targets for future therapies in the neuropsychiatric field.

## 1. Introduction

Major depressive disorder (MDD) is a prevalent neuropsychiatric disorder. Despite the effort to elucidate the neurobiology of MDD, its pathophysiology remains poorly understood, and the available therapeutic compounds are not effective in every patient. Therefore, it is crucial to fully unravel the mechanisms underlying this disorder and discover new therapeutic targets.

Several hypotheses have been proposed to explain the neurobiological mechanisms underlying the onset, maintenance, and recovery of depression [1,2,3,4]. During the last decades, a significant number of studies revealed cell loss and neuronal atrophy, particularly in brain loci relevant for emotional behavior control—the hippocampus [5,6,7,8,9,10,11]. Multiple mechanisms were proposed to be responsible for this neuronal atrophy, namely glucocorticoid and glutamate toxicity for both astrocytes and neurons [12], decreased expression of neurotrophic factors [13,14], and decreased neuroplasticity [15,16,17]. However, most of the evidence gathered to date focuses on neuronal cells in disregard of glial cells, which has, for a long time, contributed to a neurocentric view of the disease. It is now well recognized that glial cells, namely astrocytes, are not only responsible for providing support to neuronal cells, but they also undergo several plastic alterations both in the healthy and depressed brain [8,18,19,20,21,22,23]. Astrocytes are the most common type of glial cells in the adult mammalian brain, playing a relevant role in neuronal activity and function modulation [24,25,26,27]. Astrocytes are being recognized as a stress response hub, influencing cytogenesis, morphology, and synaptic plasticity in chronic stress contexts, and other diseases [28,29,30,31,32,33]. Studies either in animal models of depressive-like behavior [34,35,36,37,38] or in postmortem brain tissue of MDD patients [8,39,40,41] have reported a decreased number of astrocytes in different frontolimbic areas, including the medial prefrontal cortex (PFC), as well as in the dorsolateral and orbitofrontal cortex, the amygdala, and the hippocampus. Furthermore, the expression of S100β, a selective marker of mature astrocytes, was also found to be altered in postmortem brain tissue of depressive patients [42]. Besides cell density alterations, astrocytic size and morphology are also changed in depressed individuals. Increased glial cell nuclei size [39,41,43,44] has been observed in depressed individuals and proposed to be compensatory response to the metabolic needs of the surrounding neurons [8,39,45,46].

Importantly, astrocytes were recently described to integrate neural circuits involved in MDD, suggesting a relevant role for several emotional behaviors [47,48]. Specifically, selective deletion of astrocytes in the medial PFC induce depressive-like behavior and triggers cognitive impairments in rodents [10,49,50]. However, there is still little research on the potential of newborn astrocytes both in the pathophysiology of depression and in the actions of current antidepressants (ADs).

The generation of astrocytes—astrogliogenesis—in the adult brain has been repeatedly demonstrated [8,36,49,50,51]. Unlike neurons, glial cells retain their ability to proliferate in most areas of the brain, postnatally and even during adulthood [8,52,53,54]. Particularly, the generation of astrocytes is also detectable in the neocortex and hippocampus of the adult human brain [55,56]. Concomitantly, in adult rats, around 15% of newborn cells in the hippocampal dentate gyrus (DG) are positive for the astrocytic marker glial fibrillary acidic protein (GFAP) weeks post cell birth, using a tracing method [8]. Interestingly, glucocorticoid treatment—which mimics the elevation of blood glucocorticoids (GCs) that may occur under chronic stress exposure—decreases astrocytic proliferation in the adult rat hippocampus [57,58]. In line with this effect, in rats, exposure to chronic unpredictable stress decreases astrocyte proliferation in the prelimbic cortex [35,37]. Of relevance, this stress-induced decrease in newborn astrocytes is counteracted by the treatment with the AD fluoxetine [35,37]. Interestingly, fluoxetine treatment does not alter the neuron-to-glia ratio, suggesting it also increases the number of newborn astrocytes in the adult brain [8]. Therefore, it becomes necessary to clarify how newborn astrocytes are modulated by stress and ADs treatment, and which molecular changes are impairing adult astrogliogenesis in the context of MDD.

To better understand the importance of astrogliogenic plasticity in the context of depression, we longitudinally assessed dynamic alterations of resident and newborn astrocytes in the hippocampal DG of rats under exposure to unpredictable chronic mild stress (uCMS) and AD treatment either with fluoxetine—a selective inhibitor of serotonin reuptake—or imipramine—a tricyclic agent and a potent inhibitor of serotonin and norepinephrine reuptake. We assessed short- and long-term astrocytic alterations immediately after stress exposure and AD treatment and after a four-weeks period, respectively. We show that imipramine increased the number of newborn astrocytes in the hippocampal DG 4 weeks after stress exposure, while fluoxetine induced hypertrophy of both pre-existent and newborn astrocytes in the same region. Interestingly, only imipramine could significantly rescue the cognitive impairments transiently induced by stress exposure. Therefore, this study provides a better understanding of the role of glial cells in MDD paving the way to the comprehension of astrogliogenic factors as therapeutic targets for this disease.

## 2. Materials and Methods

### 2.1. Animals

Male Wistar–Han rats 2 months of age (Charles River Laboratories, L’Arbresle, France) were used for all in vivo experiments. Those animals were housed in groups and kept under standard laboratory conditions, i.e., 22 ± 1 °C, 12 h light/dark cycle, 55% relative humidity, and ad libitum access to water and food. Rats from three independent cohorts were randomly divided into four groups (*n* = 14–16 per group were used for behavioral analysis, of which 6–8 were used for gene expression quantification, immunofluorescence, and morphologic studies. In detail, we used samples from three independent cohorts for a short-term analysis (immediately after stress exposure—tp1) and samples from two independent cohorts for a long-term analysis (4 weeks after stress exposure—tp2), for gene expression quantification, immunofluorescence, and morphologic purposes. All the procedures were conducted under the European Directive 2010/63/EU, and experiments were approved by the University of Minho Subcommittee of Ethics for the Life and Health Sciences (SECVS068/2017).

### 2.2. Unpredictable Chronic Mild Stress—uCMS- and Drug Treatment

Animals were exposed to a pre-validated 6-weeks uCMS protocol, as already described [15,16]. This protocol induced depressive-like behavior, anxious phenotype, as well as cognitive deficits in rats through an arbitrary and unpredictable exposure to various different mild stressors. Following previous studies [15,16], in the last 2 weeks of the uCMS protocol, animals were daily injected with saline (SAL, 0.9% NaCl, intraperitoneal injection) or with distinct Ads—fluoxetine (FLX; 10 mg·kg^−1^, Kemprotec, Middlesbrough, UK) or imipramine (IMIP; 10 mg·kg^−1^; Kemprotec). Concomitantly, a group of animals was not exposed to uCMS but was injected with saline (CTRL). 

To track adult-born cells formed immediately after ADs treatment, all groups of animals received injections of bromodeoxyuridine (BrdU, 50 mg kg^−1^; intraperitoneal injections; Sigma-Aldrich, St. Louis, MO, USA) for 5 days (2 days during and 3 days next to the termination of the uCMS protocol). A subset of animals (*n* = 8–10) was not subjected to any stressor in the following 4 weeks after uCMS exposure (long-term time point of analysis). 

### 2.3. Behavioral Analysis

During the experimental protocol, we monitored rats’ behavior at different time-points. At week 6, which corresponds to the end of uCMS protocol and AD treatment, animals were submitted to the Sucrose Consumption test to assess anhedonic-like behavior [59], to the Forced Swimming test to measure stress-coping [59], and to the Open-field test to evaluate anxiety-like behavior. To assess memory performance in a longitudinal manner, rats were subjected to the Novel Object Recognition test immediately after uCMS and 4 weeks later. 

#### 2.3.1. Forced Swim Test (FST)

FST trials were performed 24 h after a 5 min pre-test session. For this purpose, rats were placed in water filled glass cylinders (23 °C; 50 cm deep) during 5 min. An increase in immobility time was related with an impaired performance to cope with an inescapable stress.

#### 2.3.2. Sucrose Consumption Test (SCT)

Rats were exposed to a sucrose solution in the week before the start of the uCMS protocol with the aim of establishing the baseline preference levels. To assess sucrose preference, access to food and water was denied for 12 h, after which the animals were presented with two pre-weighed bottles—one containing 2% sucrose solution and the other tap water—for a period of 1 h (starting at the beginning of the dark period). Sucrose preference calculation was considered as previously described [15,59].

#### 2.3.3. Open Field Test 

Open-field test was performed in a room brightly illuminated by white light. Briefly, rats were placed in the center of an arena (43.2 × 43.2 cm^2^, transparent acrylic walls and white floor, MedAssociates, St Albans, VT, USA), and their position was monitored over 5 min through two 16-beam infrared arrays. The percentage of time spent in the center of the arena was used as a direct measure of anxiety-like behavior. 

#### 2.3.4. Novel Object Recognition (NOR) 

Short and long-term memory were assessed through the NOR test [60]. To first familiarize the rats with the testing arena that consisted of a black acrylic box (50 cm × 50 cm × 150 cm) with an open field space, they were placed inside it for 8 min without any objects. On the day after, rats were allowed to freely explore the arena with two identical objects, for 10 min. Then, 24 h later, the animals returned to the arena for 3 min, where one of the objects was replaced by a new one. Importantly, the familiar and new objects were different in color, shape, size, and texture. Between trials, the arena was always cleaned with 10% ethanol to avoid odor cues [61]. All sessions were videotaped and the time spent exploring each object was assessed manually (blind analysis). For repeated testing, different objects were used at each time point (tp1 and tp2). The percentage of time spent exploring the novel object was correlated with long- and short-term memories performance.

### 2.4. Serum Corticosterone Levels Measurement

Corticosterone levels were measured in the rat’s blood serum using a radioimmunoassay (RIA) kit (MP Biomedicals), according to the manufacturer’s instructions. Blood sampling (tail venipuncture) was performed at the beginning of the diurnal period (Nadir, N, 08:00–09:00) and of the nocturnal period (Zenith, N, 20:00–21:00) in the sixth week of the uCMS protocol [62].

### 2.5. Hippocampal DG Primary Cultures

Three- to five-day-old rats (Wistar Han) were rapidly decapitated and their brains collected. Then, we removed the meninges, separated the hemispheres, and macrodissected the hippocampi in ice-cold DMEM + 10% FBS. After mechanical trituration and several washes in DMEM + 10% FBS, cells were seeded in 12-well plates (NUNC) with neurospheres medium (3 mL; DMEM-F12-GlutaMAX™, B27 supplement 2%, Pen-Strep 1%, HEPES buffer 8 mM, bFGF, and EGF (10 ng/μL). We added Dexamethasone (DEX, Fortecortin, Merck; 1 μM) to all plates besides the control ones. Viable progenitor hippocampal DG cells were counted by trypan blue exclusion assay in a hemocytometer and platted in PDL-coated 24-well plates at a density of 80,000 cells–100,000/well. Cells were kept at 37 °C in 5% CO_2_ and humid atmosphere. On the following day, the plates were carefully washed 3 times with differentiation medium (Neurobasal A, B27 supplement 2%, Pen-Strep 1%, HEPES buffer 8 mM), and 500 µL of the medium was added to every plate. DEX was added every 2 days to the medium. In the last 4 days of culturing, Desipramine (10 µM, Sigma-Aldrich, St. Louis, MO, USA) or Norfluoxetine hydrochloride (10 µM, Sigma-Aldrich, St. Louis, MO, USA) were added to the culture. BrdU (10 µM, Sigma-Aldrich, St. Louis, MO, USA) was also added in the last 4 days. Cells were fixed at day 8 for further immunostaining analysis.

### 2.6. Immunostaining Procedures

#### 2.6.1. In Vivo

Rats (*n* = 6–8 per group) were deeply anesthetized with sodium pentobarbital (20%; 100 mg·kg^−1^, Eutasil^®^, Sanofi, Gentilly, France) and transcardially perfused with 0.9% saline followed by cold 4% paraformaldehyde (PFA). Animals’ brains were removed and fixed in 4% PFA, followed by a cryoprotection in 30% sucrose overnight, and finally embedded in Optimal Cutting Temperature compound (OCT, ThermoScientific, Waltham, MA, USA), snap-frozen and stored at −20 °C. Coronal sections (20 μm) containing the dorsal pole of the hippocampal dentate gyrus (DG) were further stained to measure the number and assess the morphology of astrocytic populations. Sections were stained with anti-GFAP (#20334; 1:200; Dako, Glostrup, Denmark), anti-BrdU (#6226; 1:100; Abcam, Cambridge, UK), and anti-S100β (#AMAB91038; 1:100; Dako, Glostrup, Denmark) antibodies. 4′,6-diamidino-2-phenylindole (DAPI, 1:200; Sigma Aldrich) was used for cell nuclei staining. The quantification of cells per DG was performed individually for each DG, and the density of each cell population was determined by the ratio between the total number of cells and the respective area. Double-stained (GFAP^+^BrdU^+^) cells were analyzed the same way. Analysis and cell counting were performed with a confocal microscope (Olympus FluoViewTM FV1000, Hamburg, Germany) and each area was determined with an optical microscope (Olympus BX51). Importantly, GFAP^+^ cells in DG subgranular layer that exhibited a radial morphology were not included in the analysis as these cells are typically classified as (type-1) neural stem cells. All the analyses were blind. Cell densities are conveyed as the number of cells per 100 µm^2^.

#### 2.6.2. In Vitro

All in vitro cultures were fixed in 4% PFA for 10 min at RT and then washed with PBS. PBS-T 0.5% was used to permeabilize cellular membranes for 10 min. Incubation with primary antibodies for GFAP (#20334; Dako; 1:200) or BrdU (#6226; 1:100; Abcam, 1:50) was executed during overnight at 4 °C. Secondary antibodies (1:1000; #A28175; anti-mouse Alexa-fluor^®^ 488; #A21069 anti-rabbit Alexa-fluor^®^ 568; Life Technologies, Thermo Fisher Scientific) were incubated for 2 h at RT. DAPI (Invitrogen) incubation was performed during 10 min to allow cell nuclei labelling. Slides were further washed with PBS 1× and mounted with PermaFluor mountant medium (Thermo Scientific, Waltham, MA, USA). Sections were analyzed using an Olympus BX-61 Fluorescence Microscope (Olympus, Tokyo, Japan).

For specific astrocytic genesis analysis, BrdU/GFAP double-positive cells were counted. Three coverslips and ten randomly selected microscope fields per condition were analyzed. Results are shown as the average number of GFAP^+^ or GFAP^+^BrdU^+^ cells per DAPI.

### 2.7. Morphological Analyses

To analyze astrocytic morphology, we applied the previously described open-source tool Simple Neurite Tracer of ImageJ [63], enables the tridimensional reconstruction of main astrocytic processes in GFAP-stained sections. This marker specifically stains main astrocyte processes, and its expression is tightly related to morphological alterations. After immunostaining, z-stacks of confocal images (magnification: 40×; numerical aperture: 0.65; z-interval: 0.5; image resolution: 2048 × 2048 pixels; *n* = 10–15 astrocytes per subregion/animal) were used to determine the total processes length (in µm) of each astrocyte analyzed. This analysis was performed for different DG subregions including the granule cell layer (GCL), the subgranular zone (SGZ), defined as the three deepest rows of granule cells, the inner molecular layer (IML), and the hilus. Further, only astrocytes that revealed complete processes were considered for analysis.

### 2.8. RT-PCR Measurements

For dorsal DG macrodissection, rats were firstly anesthetized with pentobarbital (20%; 100 mg·kg^−1^, Eutasil^®^, Sanofi) and transcardially perfused with 0.9% saline. Immediately after dissection, tissues were frozen and stored at −80 °C until analysis.

Total RNA from the macrodissected DGs was isolated according to the manufactures’ instructions using the Direct-Zol™ RNA Mini-Prep (Zymo Research, Irvine, CA, USA. The extracted total RNA (500 ng) was reverse-transcribed using qScript cDNA SuperMix (Quanta Biosciences, Gaithersburg, MD, USA).

For real-time (RT)-PCR, oligonucleotide primers for S100 calcium-binding protein β (S100β, sense CACCGACTGGGCAAAATACT, antisense TCCGAACTTCCATGTCC), Glial Fibrillary Acidic Protein (GFAP, sense GGACCAGCTTACTACCAACAGTGCC, antisense TGGTTTCATCTTGGAGCTTCTGCCT), Signal transducer and activator of transcription 3 (STAT3, sense TGGACCGTCTGGAAAACTGGATAAC, antisense CTCCACCACGAAGGCACTCTTCATTA), Bone morphogenetic protein 4 (BMP4, sense TCCATCACGAAGAACATCTGGAGAA, antisense GTCCACCTGCTCCCGAAATAGC) jmjd3 (sense CGGTTCTGCCCAGTCTGTGAAACCG, antisense ATGCTGGGTGTAGGAGGGTTG), and B2M (sense GTGCTTGCCATTCAGAAAACTCC, antisense AGGTGGGTGGAACTGAGACA) were designed using the Primer-BLAST software (NCBI). Reactions were performed in an Applied Biosystems 7500 Fast Real-Time PCR System (Applied Biosystems, Waltham, MA, USA) using 5× HOT FIREPol EvaGreen qPCR Mix Plus, ROX (Solis BioDyne, Tartu, Estonia). Target gene expression levels were normalized against the housekeeping gene Beta-2-Microglobulin (B2M). The relative expression was determined using the ddCt method, and the results are presented as fold-change of mRNA levels between the respective experimental groups after normalization to B2M levels.

### 2.9. Statistical Analysis

Statistical analysis was completed using Prism 8.0 (GraphPad Software, Inc., La Jolla, CA, USA). The assignment of the animals to the experimental groups was performed randomly. All presented data fulfilled normal distribution in Kolmogorov–Smirnov testing and were subjected to the appropriate statistical tests (after confirmation of the homogeneity of group variances). Student’s *t*-test was used for statistical comparisons between experimental groups when appropriate. The comparison between stressed groups was assessed using one-way analysis of variance. Analysis of variance repeated measures was used to analyze the number of intersections from the soma. Descriptive statistical results are presented as mean ± standard error of the mean (SEM). Differences between groups were determined by Bonferroni’s post hoc multiple comparison tests, and statistical significance was set at *p* < 0.05. 

## 3. Results

### 3.1. Imipramine Induces the Generation of New Astrocytes in the Hippocampal Dentate Gyrus

**Figure 1:**
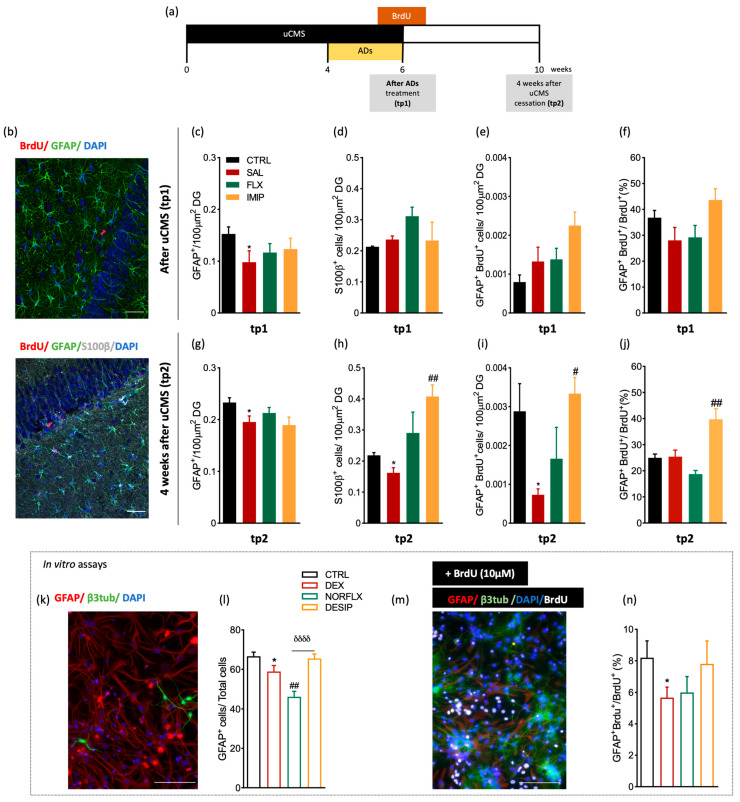
In vivo longitudinal analysis of astrocytic markers in the dorsal hippocampal dentate gyrus (dDG) of an animal model of depression and in in vitro primary cultures. (**a**) Illustrative experimental timeline. (**b**) Hippocampal DG coronal section immunostained for bromodeoxyuridine (BrdU) (in red), glial fibrillary acidic protein (in green), and DAPI (in blue). Additionally, a hippocampal DG coronal section immunostained for BrdU (in red), GFAP (in green), S100β (in grey), and DAPI (in blue). Quantitative analysis of GFAP^+^ cells in the dorsal dentate gyrus (dDG) at tp1 (**c**) and at tp2 (**g**), after a six-week uCMS protocol including antidepressants (ADs) treatment, fluoxetine, and imipramine. (**d**,**h**) Quantitative analysis of the number of S100β+ cells in the dDG, both at tp1 (**d**) and tp2 (**h**). (**e**,**i**) Quantitative analysis of GFAP+BrdU+ cells, both immediately after stress exposure, tp1, (**e**) and 4 weeks after stress exposure, tp2 (**i**). (**f**,**j**) Analysis of the number of GFAP^+^BrdU^+^ cells per total number of BrdU+ cells, both at tp1 (**f**) and at tp2 (**j**). (**k**,**m**) Representative image of immunocytochemistry of hippocampal primary cells in a control plate (**k**), and incubated with BrdU (**m**), with neurons labelled with β3-tubulin, astrocytes with GFAP proliferating cells with BrdU and cell nucleus with DAPI. (**l**,**n**) In vitro analysis of hippocampal DG primary cultures of p3–5 animals, regarding the number of GFAP^+^ astrocytic cells (**l**) and astrocytes differentiation—GFAP^+^BrdU^+^ (**n**) after incubation of the primary cell cultures with dexamethasone (DEX), norfluoxetine (NORFLX), or desipramine (DESIP) and BrdU. * Represents uCMS effect analyzed by Student’s *t*-test. # Represents ADs effect, by comparison of treatment and SAL animals; δ represents differences between ADs, analyzed by one-way analysis of variance (ANOVA). Data are represented as mean ± s.e.m. Scale bars represent 50 μm.*, # *p* < 0.05, ## *p* < 0.01, δδδδ *p* < 0.0001; Sample size: TP1: CTRL: 5–7; CMS: 5–7; FLX: 6–8; IMIP: 4–7; TP2: CTRL: 6–8; CMS: 6–9; FLX:6–8; IMIP: 6–8. Abbreviations: GFAP, Glial Fibrillary Acidic Protein; CTRL, non-stressed animals; SAL, animals exposed to uCMS and injected with saline; IMIP, animals exposed to uCMS and treated with imipramine; FLX, animals exposed to uCMS and treated with fluoxetine; dDG, dorsal dentate gyrus; DEX, dexamethasone; NORFLX, Norfluoxetine; DESIP, Desipramine; β3tub, β3-tubulin; DAPI, 4′,6′-diamino-2-fenil-indol; BrdU, Bromodeoxyuridine; tp1, time point 1 (6 weeks; immediately after the stress protocol cessation); and tp2, time point 2 (10 weeks; 4 weeks after the stress protocol cessation).

First, we assessed in a longitudinal manner the impact of chronic stress and ADs treatment on the number of existing and de novo population astrocytes in the hippocampal DG. The density of pre-existent astrocytes was assessed by the density of GFAP^+^ cells (that includes immature and mature astrocytes or S100β^+^ cells). To quantify newborn astrocytes, animals were injected with BrdU [64] 2 days before and 3 days after cessation of the uCMS protocol and ADs administration (Figure 1a,b). Assessment of astrocytic number in the dorsal dentate gyrus (dDG) immediately after chronic stress exposure (tp1) revealed no major differences between control and uCMS exposed groups, either on GFAP^+^ cells population (Figure 1c), on S100β^+^ cells (Figure 1d), and on GFAP^+^BrdU^+^ cells (Figure 1e,f). 

However, at 4 weeks after the end of the uCMS protocol—tp2—we observed a significant decrease in the number of GFAP^+^ cells promoted by uCMS (*p* = 0.04, *t*_(12)_= 1.92; Figure 1g), as supported by previous studies [38,65]. Treatment with fluoxetine or imipramine did not reverse uCMS-induced changes (Figure 1g). Regarding mature astrocytes—labeled with S100β [66]—chronic stress exposure induced a reduction in the number of S100β^+^ cells in the hippocampal DG at tp2 (*p* = 0.04, *t*_(11)_= 3.04; Figure 1h). Imipramine, but not fluoxetine, treatment significantly increased the number of S100β^+^ cells, to higher levels compared to those presented by the control group (*p* = 0.002, *F*_(2,31)_= 10.29; Figure 1h). Furthermore, when exploring the effect of stress and ADs on newborn astrocytes at tp2, a time-point at which newborn cells should have started differentiating and integrating into the circuitry [16], chronically stressed animals presented a significant reduction in newborn astrocytes (*p* = 0.02, *t*_(11)_= 2.96; Figure 1i). Interestingly, imipramine treatment elicited a strong pro-astrogliogenic response with an higher number of both GFAP^+^BrdU^+^ cells (*p* = 0.02, *F*_(2,29)_= 6.16; Figure 1i) and GFAP^+^BrdU^+^/BrdU^+^ (*p* = 0.003, *F*(_2,33_) = 13.99; Figure 1j) in comparison to those presented by rats exposed to uCMS. Treatment with fluoxetine did not exert any alterations on the density of these cells, which is suggestive of a more pro-neurogenic response previously observed [16]. To verify if this effect was specific to newborn mature astrocytes and not to glial-like precursor cells, we analyzed the effect of stress and ADs on the number of GFAP^+^S100β^+^ cells among all BrdU^+^ cells (Figure S1). No differences were found between groups in the number of GFAP^+^S100B^+^/BrdU^+^ cells at tp1 (Figure S1a). However, at tp2, stress exposure decreased the number of the newborn mature astrocytes (*p* = 0.02, *t*_(4)_= 3.31; Figure S1b) and, although not significantly different, imipramine treatment showed a tendency to increase the density of these cells (Figure S1b).

Furthermore, we analyzed the in vitro differentiation of astrocytes using primary hippocampal cell cultures from p3–5 rats. We conditioned the cells with dexamethasone (DEX) and with the active metabolites of the ADs used in the in vivo experimental approach, norfluoxetine and desipramine. By labeling the cells with GFAP and β3-tubulin antibodies (Figure 1k), after 8 days in vitro, we showed that DEX significantly decreases the number of astrocytes (GFAP^+^ cells; *p* = 0.02, *t*_(21)_ = 2.14; Figure 1l), which are restored to levels similar to the control group after desipramine conditioning, but not with norfluoxetine (*p* < 0.0001, *F_(_*_2,33)_ = 14.30; Figure 1l). Moreover, analyses of the number of GFAP^+^/BrdU^+^ cells, revealed that DEX reduces the number of newborn astrocytes (*p*= 0.0332, *t*_(9)_= 2.088; Figure 1n). Hippocampal cells treated with desipramine, but not with norfluoxetine, present a number of newborn astrocytes similar to the control untreated cells (*p* > 0.10, *F*_(2,11)_ = 1.183; Figure 1n).

### 3.2. Expression of Astrocytes’ Mediator Factors

**Figure 2:**
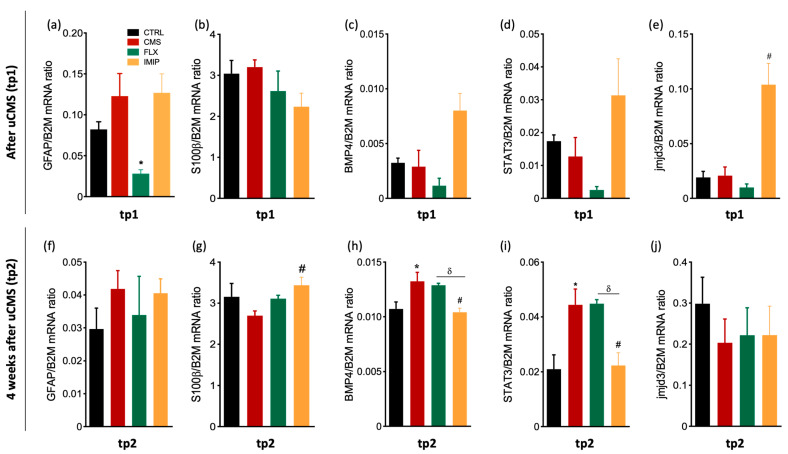
Relative mRNA expression levels of astrocytic and astrogliogenic related genes at tp1 and tp2 in the macrodissected DG. We analyzed the mRNA expression levels of GFAP (**a**,**f**), S100β (**b**,**g**), BMP4 (**c**,**h**), STAT3 (**d**,**i**), and jmjd3 (**e**,**j**) in macrodissected dentate gyrus tissue from control animals and uCMS-exposed animals treated either with saline, fluoxetine, or imipramine at tp1 and tp2. * Represents uCMS effect analyzed by Student’s *t*-test; # Represents ADs effect, by comparison of treatment and SAL animals, analyzed by one-way analysis of variance (ANOVA); and δ represents differences between ADs, analyzed by one-way analysis of variance (ANOVA). Data are represented as mean ± s.e.m. *, #, δ *p* < 0.05; Sample size: TP1: CTRL: 3–4; CMS: 4–6; FLX: 3–5; IMIP: 3–5; TP2: CTRL: 3–4; CMS: 3–4; FLX: 3–4; and IMIP: 3–4. CTRL, non-stressed animals; IMIP, animals exposed to uCMS and treated with imipramine; FLX, animals exposed to uCMS and treated with fluoxetine; SAL, animals exposed to uCMS and injected with saline; GFAP, Glial Fibrillary Acidic Protein; S100β, S100 calcium-binding protein β; BMP4, Bone morphogenetic protein 4; STAT3, Signal transducer and activator of transcription 3; JMJD3, histone H3 Lys 27 (H3K27) demethylase; tp1, time point 1 (6 weeks; immediately after the stress protocol cessation); and tp2, time point 2 (10 weeks; 4 weeks after the stress protocol cessation).

Next, we quantified the levels of several genes expressed by resident astrocytes, such as GFAP and S100β, and other genes related to astrocytic differentiation, such as BMP4, STAT3, and JMJD3. We show that, at tp1, both GFAP and S100β expression levels are not significantly changed by chronic stress exposure (GFAP: *p* = 0.2344, *t*_(4)_ = 1.399; S100β: *p* = 0.3673; *F*_(2,11)_ = 1.764, *p* = 0.2166, Figure 2a,b). However, there was a significant decrease in GFAP expression after fluoxetine administration (*p* = 0.0397, *F*_(2,7)_ = 6.220; Figure 2a). Analyses of the genes associated with astrocytic differentiation revealed that, despite no significant alterations induced by stress exposure (BMP4: *p* = 0.8308, t_(4)_ = 0.228; Figure 2c; STAT3: *p* = 0.5799, *t_(_*_4)_ = 0.618, Figure 2d; and jmjd3: *p* = 0.8955, t_(4)_ = 0.1428 Figure 2e), treatment with imipramine treatment induces a tendency for increased expression levels of BMP4 and STAT3 (BMP4: *p* = 0.0501, *F* = 5.780, Figure 2c; STAT3: *p* = 0.076, *F*_(2,5)_ = 4.063, Figure 2d) and significantly increases JMJD3 when compared to the stress-exposed group (*p* = 0.0095, *F*_(2,5)_ = 13.60; Figure 2e). 

At tp2, no differences were found on GFAP expression levels between control and stress groups (*p*= 0.2224; *t*_(5)_ = 1.443, Figure 2f) and upon treatment with ADs (*p* = 0.7600, *F*_(2,6)_ = 0.2874; Figure 2f). Interestingly, chronic stress exposure did not induce statistically significant changes in S100β expression, but imipramine treatment was able to increase S100β expression levels by comparison to the stress-exposed group (CTRL vs. CMS: *p*= 0.2535; ADs treatment: *p* = 0.0227, *F*(2,5) = 8.858; Figure 2g). Curiously, BMP4 and STAT3 expression levels are increased in the uCMS-exposed group, in comparison to the control group (BMP4: *p*= 0.0512, *t*_(6)_ = 2.429, Figure 2h; *STAT3*: *p*= 0.0399, *t*_(4)_= 3.002, Figure 2i). Plus, we observed a decreased expression of these genes in the rats treated with imipramine, when comparing to the stress-exposed group (BMP4: *p* = 0.0089, *F*_(2,9)_ = 8.353, Figure 2h; STAT3: *p*= 0.0169, *F*_(2,6)_= 8.697, Figure 2i). The expression levels of JMJD3 were not changed among groups at tp2, neither promoted by stress exposure (*p* = 0.3344, *t*_(a)_= 1.097; Figure 2j) nor treatment with ADs (*p* =0.9737, *F*_(2,6)_ = 0.0268; Figure 2j).

### 3.3. Fluoxetine Induces Hypertrophy of Resident and Newborn Astrocytes in the Hippocampal Dentate Gyrus

A large body of evidence has consistently reported that depression and stress significantly impact on morphometric properties of astrocytes, including in the size and branching (reviewed in [67] either in animal [34,65,68] and human studies [41,69]. Therefore, we sought to understand the impact of chronic stress and treatment with Ads on astrocytic morphology in the hippocampal DG. 

**Figure 3:**
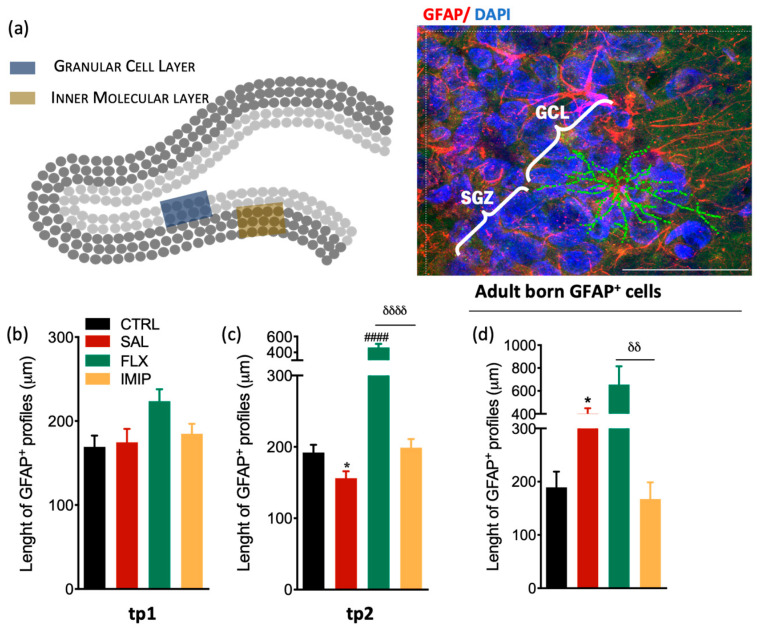
Morphological analysis of resident and newborn astrocytes in the hippocampal DG in a rat model of depression and after antidepressant treatment. (**a**) Representative scheme of the hippocampal DG regions where astrocytes were analyzed (left panel) and representative immunostaining and morphological analysis of GFAP^+^ cells in the hippocampal DG (right panel). (**c**,**d**) Longitudinal determination of the astrocytic length in the dorsal hippocampal dentate gyrus (dDG) in an experimental animal model of depression, at tp1 (**b**) and tp2 (**c**), specifically from GCL and IML. (**d**) Evaluation of astrocytic length in the granular cell layer (GCL) of the hippocampal DG newborn astrocytes, 4 weeks after cessation of the uCMS protocol and after treatment with fluoxetine and imipramine. These cells were identified by co-labeling GFAP^+^ and BrdU^+^ and were selected in the GCL to avoid stem cell analysis. * Represents uCMS effect analyzed by Student’s *t*-test. δ represents differences between ADs, analyzed by one-way analysis of variance (ANOVA). Data are represented as mean ± s.e.m. Scale bar represents 100 μm.* *p* < 0.05, δδ *p* < 0.01, ####, δδδδ *p* < 0.0001. Sample size: TP1: CTRL: 7; CMS: 10; FLX: 7; IMIP: 7; TP2: CTRL: 7; CMS: 10; FLX: 3–4; IMIP: 3–4; Adult-born GPAF^+^: CTRL: 5; CMS: 7; FLX: 5; and IMIP: 4. Abbreviations: GFAP, Glial Fibrillary Acidic Protein; CTRL, non-stressed animals; SAL, animals exposed to uCMS and injected with saline; IMIP, animals exposed to uCMS and treated with imipramine; FLX, animals exposed to uCMS and treated with fluoxetine; dDG, dorsal dentate gyrus, SGZ, subgranular zone; GCL, granule cell layer; tp1, time point 1 (6 weeks; immediately after the stress protocol cessation); and tp2, time point 2 (10 weeks; 4 weeks after the stress protocol cessation).

We assessed the morphology of astrocytes (GFAP^+^) from different sub-sections of the DG, in the granular cell layer (GCL) and inner molecular layer (IML), and presented the results together (Figure 3a). We did not include astrocytes from the subgranular zone (SGZ), to avoid the inclusion of stem cells (which are also GFAP^+^) in the analysis, which may be misleading. We found that, immediately after stress exposure, at tp1, astrocytic morphology is not altered neither by stress exposure (*p* = 0.7983, *t*_(25)_ = 0.2583, Figure 3b) nor by ADs treatment (*p* = 0.0384, *F*_(2,53)_ = 3.469; Figure 3b). However, at tp2, we observed decreased processes length in astrocytes from rats initially exposed to chronic stress in comparison to the control group (*p* = 0.0302, *t*_(23)_ = 2.352 Figure 3a). Additionally, astrocytes from fluoxetine-treated animals presented their processes length around two times higher than CMS animals (*F*_(2,36)_ = 49.31, *p* < 0.0001; Figure 3a), suggesting that this AD promotes hypertrophy of astrocytes upon stress exposure. Moreover, to understand the impact of chronic stress and ADs treatment on the morphology of newborn astrocytes, we analyzed the morphology of GFAP^+^BrdU^+^ cells in the GCL of the hippocampal DG at tp2. Newborn astrocytes from uCMS-exposed animals presented increased processes length when compared to astrocytes from the control group (*p* = 0.0108; *t*_(17)_ = 3.201 Figure 3d) similar to fluoxetine-treated animals, while imipramine-treated animals revealed astrocytes with processes length equivalent to control astrocytes (*p* = 0.0052, *F*_(2,20)_ = 8.802; Figure 3b).

### 3.4. Imipramine, but Not Fluoxetine, Rescues Cognitive Impairments Induced by Stress Exposure

**Figure 4:**
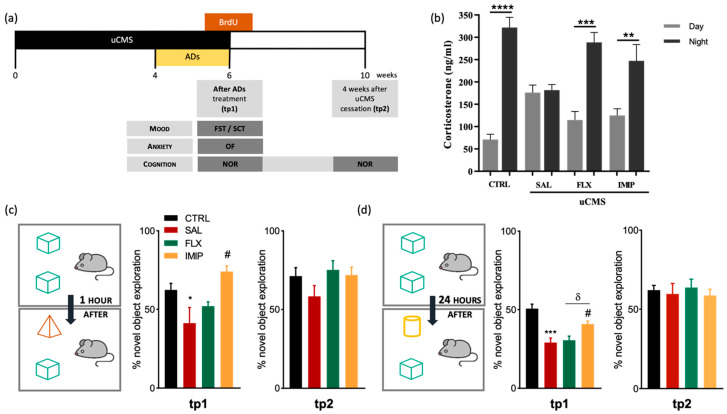
Treatment with imipramine rescues short-term and long-term memory deficits induced by chronic stress. (**a**) Representation of the current experimental timeline, including behavioral assessments and treatments. (**b**) Evaluation of corticosterone levels in rats blood serum, collected at nadir and zenith timepoints, at tp1 (immediately after the end of the uCMS protocol) (**c**,**d**) Evaluation of short- (**c**) and long-term memory (**d**) in the novel object recognition test (NOR), both at tp1 and tp2. * Represents uCMS effect analyzed by Student’s *t*-test; # Represents ADs effect, by comparison of treatment and SAL animals; and δ represents differences between ADs, analyzed by one-way analysis of variance (ANOVA). Data are represented as mean ± s.e.m. *, #, δ *p* < 0.05, ** *p* < 0.01, *** *p* < 0.001, **** *p<* 0.0001; Sample size: Corticosterone assay: CTRL: 12–14; CMS: 7–8; FLX: 8; IMIP: 6–10; TP1: CTRL: 10; CMS: 6; FLX: 8; IMIP: 8; TP2: CTRL: 10; CMS: 8; FLX: 8; and IMIP: 8. uCMS, unpredictable chronic mild stress protocol; AD, antidepressant; CTRL, non-stressed animals; IMIP, animals exposed to stress protocol and treated with imipramine; FLX, animals exposed to stress protocol and treated with fluoxetine; FST, forced-swimming test; NOR, novel object recognition; SCT, sucrose consumption test; SAL, animals exposed to stress protocol and injected with saline; OF, Open field test; tp1, time point 1 (6 weeks; immediately after the end of the uCMS protocol); and tp2, time point 2 (10 weeks; 4 weeks after the end of the uCMS protocol).

To validate the phenotype typically observed in rats exposed to the uCMS protocol, we analyzed their short-term effects on behavior and impact on the regulation of the HPA axis [15]. Rats exposed to uCMS protocol presented an impaired coping phenotype (as denoted by a significant increase in immobility time in the Forced Swimming Test and an anhedonic-like revealed by a lower sucrose preference in the Sucrose Consumption Test (FST: *p* = 0.0235, *t*_(15)_ = 2.56, Figure S2a; SCT: *p* = 0.0006, *t*_(16)_ = 4.40, Figure S2b). Treatment with fluoxetine and imipramine rescues these uCMS-induced behavioral changes (FST: *p* = 0.0158, F_(2,15)_ = 5.803; SCT:, *p* = 0.0081, *F*_(2,16)_ = 6.924; Figure S2a,b). Although the results did not reach statistical significance, we also observed a tendency of uCMS-exposed animals for an anxiety-like behavior in the OF test (*p* > 0.1, *t_(_*_15)_ = 1.20; Figure S1c). Furthermore, given that chronic stress leads to a hyperactivation of the Hypothalamic–Pituitary-Adrenal (HPA) axis with consequent alterations in corticosterone blood levels, and in the circadian rhythm of blood corticosterone secretion [70], we analyzed serum corticosterone levels at night and day timepoints. Assessment of corticosterone levels of chronically stressed animals revealed a disruption in the HPA axis, with similar values of corticosterone at the nadir and the zenith timepoints of analyses (*p* > 0.1, *t*_(14)_ = 0.26; Figure 4b). However, animals treated with fluoxetine and imipramine showed a similar profile to control animals, with higher levels of corticosterone at the night timepoint, suggesting that both ADs were able to re-establish the HPA axis function (CTRL: *p* < 0.0001, *t*_(24)_ = 8.30; FLX: *p* = 0.0006, *t*_(12)_ = 5.95; IMIP: *p* = 0.0049, *t*_(12)_ = 3.445; Figure 4b). 

We characterized cognitive alterations upon uCMS exposure and ADs administration in a longitudinal manner. As such, we assessed the cognitive performance of the animals in the Novel Object Recognition, testing for short- and long-term memory, immediately after exposure to stress (tp1), and four weeks after exposure to stress (tp2) (see Figure 4a for a schematic representation of the experimental timeline). We observed that at tp1, stressed animals presented short-term memory deficits (*p* = 0.0277, *t*_(12)_ = 2.24; Figure 4c) and imipramine, but not fluoxetine, treatment rescued this cognitive impairment (*p* = 0.0168, *F*_(2,10)_ = 6.318; Figure 4c). Regarding long-term memory, reported to be more dependent on hippocampal function [71], chronically stressed animals showed long-term memory deficits at tp1 (*p* = 0.0004, *t*_(13)_ = 4.67; Figure 4d), with only imipramine treatment being able to restore the long-term memory performance (*p* = 0.0110, F_(2,12)_ = 6.982; Figure 4d). Despite these cognitive impairments at short-term (tp1), at tp2 uCMS-exposed animals recovered from short-term and long-term memory deficits to the levels of control animals (short-term memory: *p* = 0.1609, *t*_(12)_ = 1.48; Figure 4c; long-term memory: *p* = 0.7172; *t*_(13)_= 0.368; Figure 4d). Furthermore, animals treated with both ADs did not exhibit any alterations at tp2, either on long-term memory or short-term memory. 

## 4. Discussion

Overall, the present study shows that two different classes of ADs have a differential impact on pre-existing and newborn astrocytes in the adult hippocampal DG in the longitudinal course of a depressive episode. Imipramine rescues depression-associated cognitive impairments and acts through astrogliogenesis potentiation, while fluoxetine induces a state of astrocytic hypertrophy on both resident and newborn astrocytes. Interestingly, this distinct effect of fluoxetine and imipramine has also been shown to occur in the context of depression recurrence, as fluoxetine, contrarily to imipramine, induced an over-production of new neurons in the hippocampal DG [61]. 

Here, we report the pro-astrogliogenic effect of imipramine in the hippocampal DG both in vivo and in vitro in response to stress conditions. Several studies have already reported the direct effect of imipramine on astrocytic differentiation, either in vivo or in vitro [16,72,73,74]. In accordance, we found that genes related to astrocytic differentiation—STAT3, BMP4, and JMJD3—were upregulated in the hippocampal DG of chronic stress-exposed animals treated with imipramine immediately after stress exposure and AD treatment (tp1). As such, it seems that there is already an effect at the molecular level at tp1, potentiating a change in cell fate that might have an impact in cognitive behavior. A fast recovery of cellular morphology induced by ADs, as observed in previous studies might be also contributing to this cognitive improvement [45,59,61]. On the other hand, the expression of these genes involved in astrocytic differentiation in fluoxetine-treated animals was similar to those exhibited by rats exposed to chronic stress at both timepoints. At least in what concerns neuronal cells development, tp1 corresponds to the onset of a slow maturation process and evaluates the role of immature neuronal cells, while at tp2 the newly formed cells attain complete maturation and functionality and are integrated into the local neurocircuitry. Therefore, those specific genes that are known to promote astrogliogenesis have their expression levels increased immediately after stress exposure and ADs treatment. However, that potentiation only leads to a significant density increase in the newborn astrocytes 4 weeks after (tp2 analysis). Importantly, the astrogliogenic effect promoted by imipramine is also corroborated by the fact that norepinephrine, in contrast to serotonin, directly activate the resident pool of progenitor cells and stimulate neurogenesis, but also gliogenesis, in vitro [75]. 

In fact, we decided to study the impact of the ADs treatment in a pool of hippocampal progenitor cells regarding astrocytic density and astrogliogenesis. As previously mentioned, chronic stress exposure can induce a disruption of the HPA axis, which results in increased GCs secretion. Therefore, to mimic this elevation of GCs after chronic stress exposure in an in vitro setting, we stimulated rat hippocampal primary cultures with dexamethasone (DEX), an agonist of GR, followed by the ADs active metabolites. Our in vitro results corroborate the in vivo findings, showing decreased generation of astrocytes after DEX treatment and a re-establishment of levels similar to the control group only after desipramine treatment. The same tendency happens when observing our results regarding astrogliogenesis in vivo, which shows the impact that chronic stress can have on astrocytes and how imipramine can revert those effects.

Furthermore, this study reports a strong impact of fluoxetine in the morphology of astrocytes. Fluoxetine increases astrocytic length and induces a state of hypertrophy in both resident astrocytes and newborn astrocytes. Astrocytes respond to several forms of CNS injury and disease through a process called reactive astrogliosis, a pathological hallmark of CNS structural lesions. Reactive astrogliosis has always been accompanied by varying degrees of cellular hypertrophy [76]. In terms of function, reactive astrocytes can absorb glutamate from the synaptic cleft, not only reducing excitotoxicity but also providing cells with the substances required for neuronal metabolism [77]. Therefore, astrocytes may be activated by fluoxetine treatment to cope with the increased neuronal production that occurs after treatment with this AD, thus responding to the network changes and assuming a protective role. 

In this study, only imipramine could efficiently rescue cognitive impairments immediately after chronic stress exposure. A previous study from our lab has shown as continuous proliferation and complete circuitry integration of new neurons and glial cells, a process that takes 4–8 weeks in rodents [78], is necessary for the maintenance of emotional and cognitive homeostasis [16]. Interestingly, imipramine could ameliorate anxiety and cognitive deficits induced by chronic stress, independently of ongoing cytogenesis, whereas the anxiolytic and pro-cognitive efficacy of fluoxetine was dependent of cytogenesis [16]. Here, we also show that cognitive impairments are fully re-established 4 weeks after stress exposure when newborn cells are already expected to be integrating in the circuits. Treatment with fluoxetine was not effective to rescue cognitive impairments in short and long-term memory, that emerge after chronic stress. Therefore, it seems that this AD is not able to exert its effect in this behavioral domain in such a short timeframe, as it acts through astrocytic morphology alterations and by increasing hippocampal neurogenesis, as previously shown [61].

Cognitive dysfunctions in MDD are a core determinant of functional impairment affecting a variety of domains such as executive function, attention, memory, processing speed, and psychomotor skills. Recent meta-analysis research on the cognitive effects of ADs in MDD found no significant differences between pharmacological classes [79]. Only multimodal ADs such as vortioxetine and the serotonin and noradrenaline reuptake inhibitor duloxetine have well-established pro-cognitive benefits in MDD [80,81,82,83]. The mechanisms of action of these drugs suggest that the noradrenergic effects of duloxetine, which tricyclic agents such as imipramine share, may be beneficial in cognitive domains, contributing to the comprehension of the differences observed in the present study between imipramine and fluoxetine. Regarding the effects of different classes of antidepressants on the differential regulation of neuro- and gliogenesis, the knowledge is, to date, very scarce. Further studies are required to unravel the contributions of different classes of ADs to the generation of distinct populations of cells, with implications for behavior modulation.

Overall, our findings suggest that complex dynamic remodeling of astrocytic networks might have a crucial role in the recovery of cognitive deficits in depression. This work shows that imipramine treatment promotes a pro-astrogliogenic response in the hippocampal DG of depressive-like animals. We hypothesize that the immediate behavioral-cognitive improvements could be related to the imipramine treatment. Moreover, fluoxetine treatment is not able to immediately rescue the cognitive impairments caused by chronic stress exposure and induces a hypertrophic effect on both resident and newborn astrocytes. Altogether, the results herein suggest that hippocampal DG resident and newborn astrocytes might constitute promising therapeutic targets for future therapies in the neuropsychiatric field. 

## Data Availability

Data is contained within the article or supplementary material.

## Supplementary Materials

The following supporting information can be downloaded at: https://www.mdpi.com/article/10.3390/cells11030390/s1, Figure S1: Representation of hippocampal DG coronal sections immunostained for bromodeoxyuridine (BrdU) (in green), glial fibrillary acidic protein (in red), and DAPI (in blue) for a visual understanding of newborn astrocytes (GFAP^+^BrdU^+^ cells); Figure S2: Representative 3D visualization of mature astrocytes analyzed with the Neurolucida software at tp1, tp2, as well as for adult born astrocytes; Figure S3: Longitudinal analysis of the number of GFAP^+^S100β^+^BrdU^+^ cells in the hippocampal dDG at tp1 (a) and at tp2 (b), after a six-week uCMS protocol that included a treatment with fluoxetine or imipramine; Figure S4: Emotional-behavior assessment immediately after the stress protocol for depressive-like phenotype validation.
