# Supplementary material for: Beyond New Neurons in the Adult Hippocampus: Imipramine Acts as a Pro-Astrogliogenic Factor and Rescues Cognitive Impairments Induced by Stress Exposure
Source: Cells. 2022 Jan 24;11(3):390. doi: 10.3390/cells11030390 (PMC8834148; doi:10.3390/cells11030390)
Supplement: Supplementary file 1 [file cells-11-00390-s001.zip › cells-1537683-supplementary.pdf]

Beyond new neurons in the adult hippocampus: Imipramine acts as a pro-astrogliogenic factor and rescues cognitive impairments induced by stress exposure

#Ana R. Machado-Santos[1,2], #Eduardo Loureiro-Campos[1,2], Patrícia Patrício[1,2], Bruna Araújo[1,2], Nuno D. Alves[1,2,α], António Mateus-Pinheiro[1,2], Joana S. Correia[1,2], Mónica Morais[1,2], João M. Bessa[1,2], Ana J. Rodrigues[1,2], Nuno Sousa[1,2], *João Filipe Oliveira[1,2,3] and *Luísa Pinto[1,2]

1 Life and Health Sciences Research Institute (ICVS), School of Medicine, University of Minho, Braga, Portugal
2 ICVS/3B's - PT Government Associate Laboratory, Braga/Guimarães, Portugal
3 IPCA-EST-2Ai, Polytechnic Institute of Cávado and Ave, Applied Artificial Intelligence Laboratory, Campus of IPCA, Barcelos, Portugal
α Current affiliation: Department of Psychiatry, Columbia University, New York, NY 10032, USA; New York State Psychiatric Institute, New York, NY 10032, USA
# These authors contributed equally to the work and are joint first authors.
* Correspondence: JFO and LP are joint corresponding authors. luisapinto@med.uminho.pt or joaooliveira@med.uminho.pt; Tel: +351253604929

Supplementary Figures:

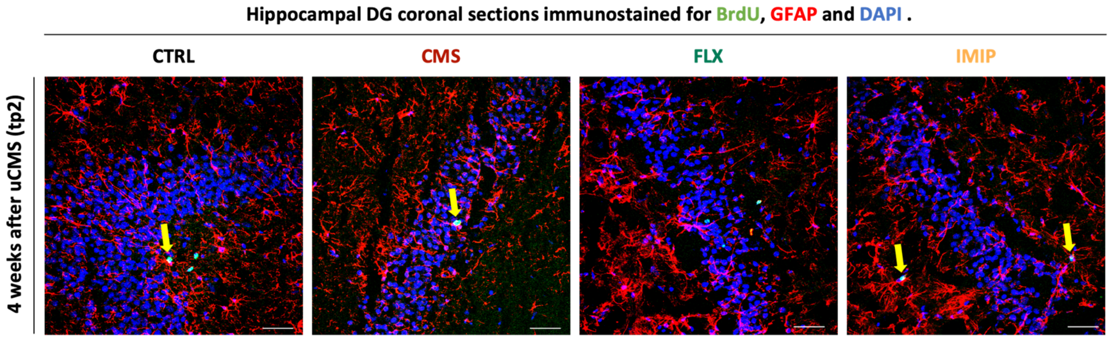

Figure S1. Representation of hippocampal DG coronal sections immunostained for bromodeoxyuridine (BrdU) (in green), glial fibrillary acidic protein (in red), and DAPI (in blue) for a visual understanding of newborn astrocytes (GFAP$^+$BrdU$^+$ cells). Images include the different conditions - control, SAL, fluoxetine, and imipramine treated animals – at time point 2. Scale bar: 50 µm. Abbreviations: DAPI, 4',6'-diamino-2-fenil-indol; BrdU, Bromodeoxyuridine; GFAP, Glial Fibrillary Acidic Protein; CTRL, non-stressed animals; SAL, animals exposed to uCMS and injected with saline; IMIP, animals exposed to uCMS and treated with imipramine; FLX, animals exposed to uCMS and treated with fluoxetine.

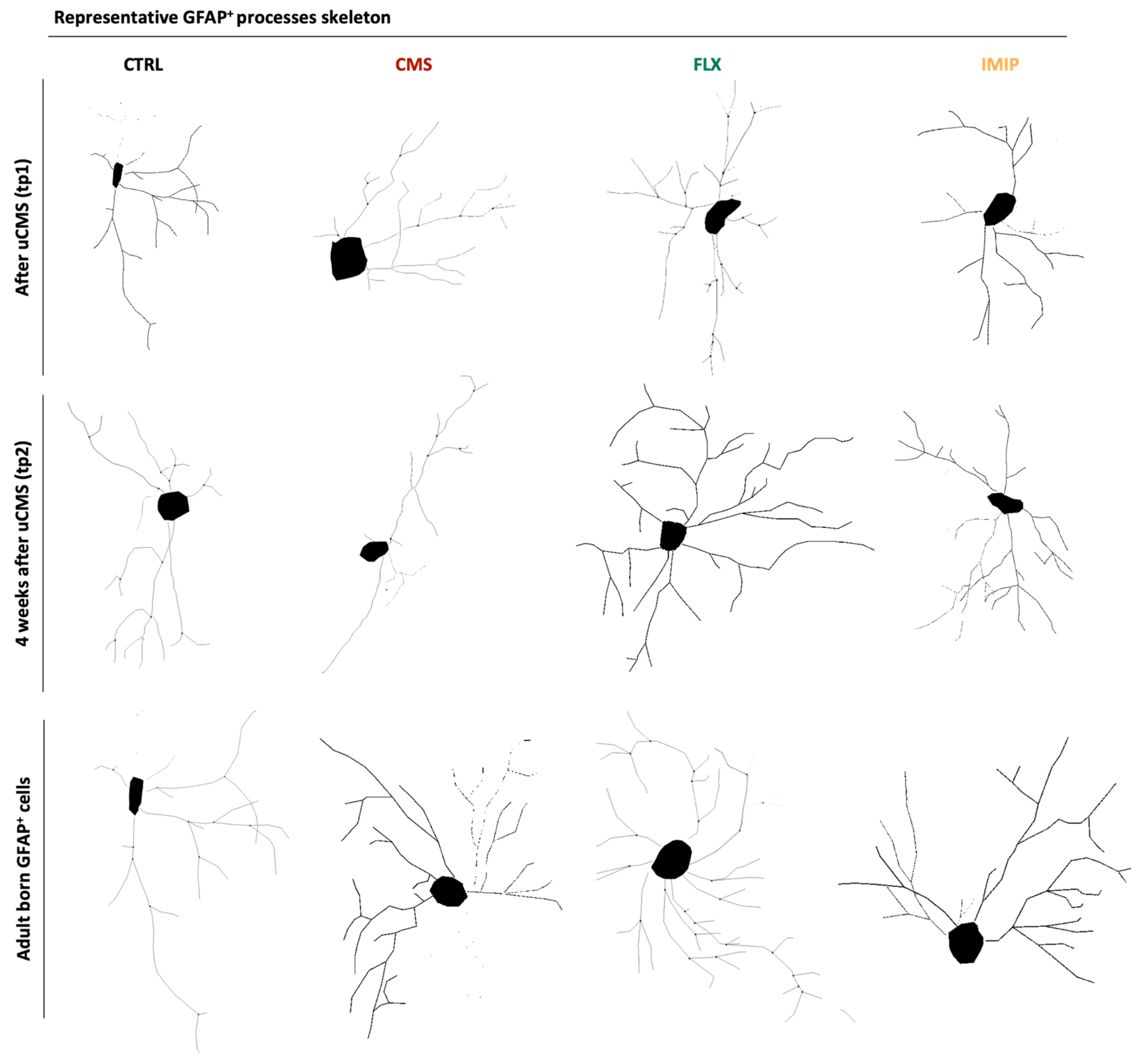

Figure S2. Representative 3D images of mature astrocytes analyzed with the Neurolucida software at tp1, tp2, as well as for adult-born astrocytes. Abbreviations: tp1, time point 1 (6 weeks; immediately after the end of the uCMS protocol); tp2, time point 2 (10 weeks; 4 weeks after the end of the uCMS protocol); GFAP, Glial Fibrillary Acidic Protein; CTRL, non-stressed animals; SAL, animals exposed to uCMS and injected with saline; IMIP, animals exposed to uCMS and treated with imipramine; FLX, animals exposed to uCMS and treated with fluoxetine.

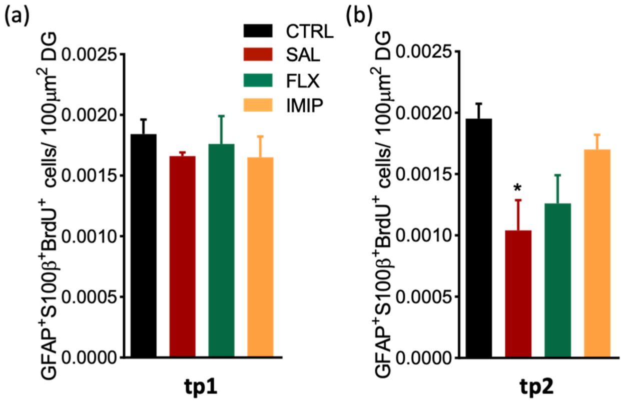

Figure S3. Longitudinal analysis of the number of GFAP$^+$S100β$^+$BrdU$^+$ cells in the hippocampal dDG at tp1 (a) and at tp2 (b), after a six-week uCMS protocol that included a treatment with fluoxetine or imipramine. *Denotes the effect of uCMS analyzed by Student's t-test. Data are represented as mean ± s.e.m. *$P<0.05$; Sample size: TP1: CTRL: 5-7; CMS: 5-7; FLX: 6-8; IMIP: 4-7; TP2: CTRL: 6-8; CMS: 6-9; FLX:6-8; IMIP: 6-8. Abbreviations: GFAP, Glial Fibrillary Acidic Protein; S100β, S100 calcium-binding protein β; CTRL, non-stressed animals; SAL, animals exposed to uCMS and injected with saline; FLX, animals exposed to uCMS and treated with fluoxetine; IMIP, animals exposed to uCMS and treated with imipramine; BrdU, Bromodeoxyuridine; tp1, time point 1 (6 weeks; immediately after the stress protocol cessation); tp2, time point 2 (10 weeks; 4 weeks after the stress protocol cessation).

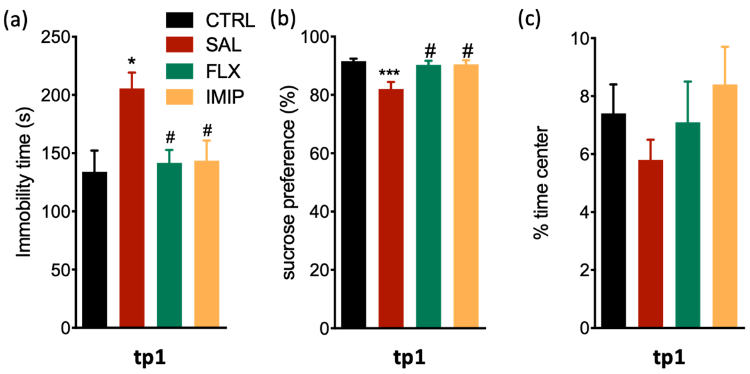

Figure S4: Emotional-behavior assessment immediately after the stress protocol for depressive-like phenotype validation. (a) Coping impaired behavior induced by chronic stress exposure was assessed by the forced swimming test (FST). (b) Anhedonic-like behavior induced by uCMS exposure was evaluated by the sucrose preference test (SPT). (c) Anxiety-like behavior was determined by the Open field test (OF). *Denotes the effect of uCMS analyzed by Student's *t*-test; #Denotes the effect of ADs, by comparison of treatment and SAL animals, analyzed by one-way ANOVA. Data are represented as mean ± SEM. *, #P< 0.05, ***P< 0.001; Sample size: TP1: CTRL: 10; CMS: 6; FLX: 8; IMIP: 8. Abbreviations: TP, time-point; CTRL, non-stressed animals; SAL, animals exposed to uCMS and injected with saline; FLX, animals exposed to uCMS and treated with fluoxetine; IMIP, animals exposed to uCMS and treated with imipramine; tp1, time point 1 (6 weeks; immediately after the stress protocol cessation).
